# The correlation between stress response term babies with intrauterine growth restriction and adulthood disease theories

**DOI:** 10.5339/qmj.2023.sqac.32

**Published:** 2023-11-26

**Authors:** Manal Eldegeir, Cornelius Ani, Sally McGregor

**Affiliations:** ^1^JACO NGO Email: almanjil@gmail.com; ^2^Division of Psychiatry, Imperial College London, London, UK; ^3^Centre for International Child Health, Institute of Child Health, University College London, London, UK

## Abstract

Background: Every year, 14 million babies are born with low birth weight (LBW) and/or intrauterine growth restriction (IUGR) in developing countries. In Sudan, 15-25% of all newborns are born with LBW, with half being Term-LBW. The importance of nutrition in the first 1000 days of life has been well demonstrated. Evidence links IUGR to various health and developmental disorders, and intrauterine programming of the hypothalamic-pituitary-adrenal (HPA) axis has been strongly suggested as a possible mechanism. Sudan has been listed among the lowest four countries regarding food security which has a massive impact on maternal and infant health.

Hypothesis: We hypothesize that infants who suffer from intrauterine growth retardation will have exaggerated physiological and behavioural responses to physical stressors.

Objectives: To compare T-LBW with T-NBW on (a) Salivary cortisol level at rest and after a physical stressor and (b) Behavioural response to physical stressors.

Methods: Hospital-based matched case-control study. Cases were 65 T-LBW neonates, and controls were 67 T-NBW neonates matched for age 4-6 hours, gestational age, and mode of deliverymeasurements: Anthropometry, salivary samples for cortisol before and after heel prick, and behavioural ratings.

Results: Compared with controls, the IUGR neonates were lighter, shorter, and thinner (p <0.0001) and had lower basal cortisol levels (p <0.03). Following stressors, IUGR neonates had lower (p >0.0001) and inhibited cortisol response (p <0.02), and cried less vigorously (p <0.0001). All anthropometric measures were significantly and positively correlated with behavioural responses and pre- and post-stress cortisol levels. Stunting was more strongly associated with behavioural inhibition than wasting.

Conclusion: The severity of intrauterine growth retardation correlated with behavioral and physiological inhibition, which can lead to the development of mismatch diseases such as allergies, autoimmune diseases, and conjunctive disorders.

